# Supplementary material for: Oral Health Status of 12-Year-Old Hani Children in the Yunnan Province of China: A Cross-Sectional Study
Source: Int J Environ Res Public Health. 2021 May 16;18(10):5294. doi: 10.3390/ijerph18105294 (PMC8156634; doi:10.3390/ijerph18105294)
Supplement: Supplementary file 1 [file ijerph-18-05294-s001.zip › ijerph-1180988-supplementary.pdf]

STROBE Statement—checklist of items that should be included in reports of observational studies

| | Item No. | Recommendation | Page No. | Relevant text from manuscript |
|---|---|---|---|---|
| **Title and abstract** | 1 | (*a*) Indicate the study's design with a commonly used term in the title or the abstract | 1 | Cross-sectional study |
| | | (*b*) Provide in the abstract an informative and balanced summary of what was done and what was found | 1 | Abstract |
| **Introduction** | | | | |
| Background/rationale | 2 | Explain the scientific background and rationale for the investigation being reported | 1-2 | Introduction |
| Objectives | 3 | State specific objectives, including any prespecified hypotheses | 2 | Aim |
| **Methods** | | | | |
| Study design | 4 | Present key elements of study design early in the paper | 2 | Cross-sectional study |
| Setting | 5 | Describe the setting, locations, and relevant dates, including periods of recruitment, exposure, follow-up, and data collection | 3 | In primary schools |
| Participants | 6 | (*a*) *Cohort study*—Give the eligibility criteria, and the sources and methods of selection of participants. Describe methods of follow-up<br>*Case-control study*—Give the eligibility criteria, and the sources and methods of case ascertainment and control selection. Give the rationale for the choice of cases and controls<br>*Cross-sectional study*—Give the eligibility criteria, and the sources and methods of selection of participants | 3 | Inclusion criteria |
| | | (*b*) *Cohort study*—For matched studies, give matching criteria and number of exposed and unexposed<br>*Case-control study*—For matched studies, give matching criteria and the number of controls per case | N/A | |
| Variables | 7 | Clearly define all outcomes, exposures, predictors, potential confounders, and effect modifiers. Give diagnostic criteria, if applicable | 3-4 | Data analysis |
| Data sources/ measurement | 8* | For each variable of interest, give sources of data and details of methods of assessment (measurement). Describe comparability of assessment methods if there is more than one group | 3-4 | Data analysis |
| Bias | 9 | Describe any efforts to address potential sources of bias | N/A | |
| Study size | 10 | Explain how the study size was arrived at | 3 | Sample size calculation |

Continued on next page

| | | | | |
|---|---|---|---|---|
| Quantitative variables | 11 | Explain how quantitative variables were handled in the analyses. If applicable, describe which groupings were chosen and why | 4 | Data analysis |
| Statistical methods | 12 | (*a*) Describe all statistical methods, including those used to control for confounding | 4 | Data analysis |
| | | (*b*) Describe any methods used to examine subgroups and interactions | N/A | |
| | | (*c*) Explain how missing data were addressed | 3 | Questionnaire survey |
| | | (*d*) *Cohort study*—If applicable, explain how loss to follow-up was addressed<br>*Case-control study*—If applicable, explain how matching of cases and controls was addressed<br>*Cross-sectional study*—If applicable, describe analytical methods taking account of sampling strategy | 4 | Data analysis |
| | | (*e*) Describe any sensitivity analyses | N/A | |
| **Results** | | | | |
| Participants | 13* | (a) Report numbers of individuals at each stage of study—eg numbers potentially eligible, examined for eligibility, confirmed eligible, included in the study, completing follow-up, and analysed | 5 | Invited, join, recruited |
| | | (b) Give reasons for non-participation at each stage | 5 | Absent from schools |
| | | (c) Consider use of a flow diagram | N/A | |
| Descriptive data | 14* | (a) Give characteristics of study participants (eg demographic, clinical, social) and information on exposures and potential confounders | Table 1 | Background information |
| | | (b) Indicate number of participants with missing data for each variable of interest | N/A | |
| | | (c) *Cohort study*—Summarise follow-up time (eg, average and total amount) | N/A | |
| Outcome data | 15* | *Cohort study*—Report numbers of outcome events or summary measures over time | N/A | |
| | | *Case-control study*—Report numbers in each exposure category, or summary measures of exposure | N/A | |
| | | Cross-sectional study—Report numbers of outcome events or summary measures | 5-6 | Prevalence rate |
| Main results | 16 | (*a*) Give unadjusted estimates and, if applicable, confounder-adjusted estimates and their precision (eg, 95% confidence interval). Make clear which confounders were adjusted for and why they were included | Table 1-2 | |
| | | (*b*) Report category boundaries when continuous variables were categorized | N/A | |
| | | (*c*) If relevant, consider translating estimates of relative risk into absolute risk for a meaningful time period | N/A | |

Continued on next page

| | | | | |
|---|---|---|---|---|
| Other analyses | 17 | Report other analyses done—eg analyses of subgroups and interactions, and sensitivity analyses | N/A | |
| **Discussion** | | | | |
| Key results | 18 | Summarise key results with reference to study objectives | 6-7 | Prevalent, not prevailed. |
| Limitations | 19 | Discuss limitations of the study, taking into account sources of potential bias or imprecision. Discuss both direction and magnitude of any potential bias | 8 | One limitaion |
| Interpretation | 20 | Give a cautious overall interpretation of results considering objectives, limitations, multiplicity of analyses, results from similar studies, and other relevant evidence | 6-7 | Whole paragraph |
| Generalisability | 21 | Discuss the generalisability (external validity) of the study results | 7 | Maintain the representativeness |
| **Other information** | | | | |
| Funding | 22 | Give the source of funding and the role of the funders for the present study and, if applicable, for the original study on which the present article is based | 8 | Funding |

*Give information separately for cases and controls in case-control studies and, if applicable, for exposed and unexposed groups in cohort and cross-sectional studies.

**Note:** An Explanation and Elaboration article discusses each checklist item and gives methodological background and published examples of transparent reporting. The STROBE checklist is best used in conjunction with this article (freely available on the Web sites of PLoS Medicine at http://www.plosmedicine.org/, Annals of Internal Medicine at http://www.annals.org/, and Epidemiology at http://www.epidem.com/). Information on the STROBE Initiative is available at www.strobe-statement.org.
